# Supplementary material for: Accuracy of Ultrasonography and Magnetic Resonance Imaging for Preoperative Staging of Cervical Cancer—Analysis of Patients from the Prospective Study on Total Mesometrial Resection
Source: Diagnostics (Basel). 2021 Sep 23;11(10):1749. doi: 10.3390/diagnostics11101749 (PMC8534714; doi:10.3390/diagnostics11101749)
Supplement: Supplementary file 1 [file diagnostics-11-01749-s001.zip › diagnostics-1341194-supplementary.pdf]

Table S1. Ontogenetic staging of cervical carcinoma [1].

Ontogenetic Tumour Stage	Adult Pelvic Tissues with Neoplastic Invasion/Intravasion
1	Mature Müllerian subcompartment: cervical epithelia and stroma
2	Mature Müllerian compartment: cervical stroma and any of the following tissues: paracervix, proximal vagina and paracolpos, corpus and paracorpus, or Fallopian tube (except fi mbria) and mesosalpinx
3	Mature genital metacompartment: Müllerian compartment and any of several tissues: ventral and dorsomedial urogenital mesentery (mesometrium, mesobladder, uterovaginal suspensory), dorsal lamina muscularis of the bladder, distal vagina, dorsal urethra, rectovaginal septum, fi mbria, ovary and mesovar, or uterine peritoneum
4	Mature urogenitorectal metacompartment: genital metacompartment and any of several tissues: bladder muscosa, ureter and mesureter, dorsolateral urogenital mesentery, anterior rectal wall and mesorectum, or pelvic peritoneum

[1] Höckel M, Hentschel B, Horn LC. Association between developmental steps in the organogenesis of the uterine cervix and locoregional progression of cervical cancer: a prospective clinicopathological analysis. Lancet Oncol. 2014;15(4):445-56. doi: 10.1016/S1470-2045(14)70060-9.

Table S2. Data for discordances calculation between imaging (ultrasonography and MRI) and histology for primary or residual tumor (after conization) measurements. (N = 50).

**Ultrasonography**					**MRI**					**Histology**					**Substitutive Diameter**			**Maximal Size**		
Dimensions					Dimensions					Dimensions										
a	b	c	$d_{s,U}$ [1]	max_U [2]	a	b	c	$d_{s,M}$ [1]	max_M [2]	a	b	c	$d_{s,H}$ [1]	max_H [2]	$\lvert d_{s,U} - d_{s,H} \rvert$	$\lvert d_{s,M} - d_{s,H} \rvert$	better [3]	$\lvert max_U - max_H \rvert$	$\lvert max_M - max_H \rvert$	better [3]
14	8	10	12.9	14	0	0	0	0.0	0	23	10	6	13.8	23	0.9	13.8	**U**	9	23	**U**
0	0	0	0.0	0	0	0	0	0.0	0	0	0	0	0.0	0	0.0	0.0	**0**	0	0	**0**
8	4	5	6.7	8	8	4	4	6.3	8	28	13	13	20.8	28	14.1	14.6	**U**	20	20	**0**
25	20	15	24.3	25	30	17	14	23.9	30	23	13	20	22.5	23	1.8	1.4	**M**	2	7	**U**
34	26	26	35.3	34	50	62	27	54.3	62	33	18	20	28.3	33	7.0	26.0	**U**	1	29	**U**
22	21	15	23.7	22	23	20	18	25.1	23	27	21	18	26.9	27	3.3	1.8	**M**	5	4	**M**
10	10	8	11.5	10	11	7	7	10.1	11	11	7	7	10.1	11	1.4	0.0	**M**	1	0	**M**
14	7	9	11.9	14	0	0	0	0.0	0	14	6	5	9.3	14	2.6	9.3	**U**	0	14	**U**
18	9	8	13.5	18	9	5	5	7.5	9	22	12	8	15.9	22	2.4	8.4	**U**	4	13	**U**
0	0	0	0.0	0	0	0	0	0.0	0	4	3	4	4.5	4	4.5	4.5	**0**	4	4	**0**
25	20	10	21.2	25	40	30	28	40.0	40	24	25	15	25.8	25	4.6	14.2	**U**	0	15	**U**
0	0	0	0.0	0	0	0	0	0.0	0	0	0	0	0.0	0	0.0	0.0	**0**	0	0	**0**
0	0	0	0.0	0	0	0	0	0.0	0	5	4	3	4.9	5	4.9	4.9	**0**	5	5	**0**
47	37	37	49.7	47	55	44	23	47.4	55	49	44	18	42.0	49	7.7	5.4	**M**	2	6	**U**
25	12	8	16.6	25	11	14	14	16.0	14	34	30	30	38.8	34	22.2	22.8	**U**	9	20	**U**
24	24	24	29.8	24	15	16	15	19.0	16	19	19	9	18.4	19	11.4	0.6	**M**	5	3	**M**
0	0	0	0.0	0	0	0	0	0.0	0	0	0	0	0.0	0	0.0	0.0	**0**	0	0	**0**
19	18	15	21.4	19	13	11	11	14.4	13	14	8	8	12.0	14	9.4	2.5	**M**	5	1	**M**
30	24	31	34.9	31	18	26	33	30.9	33	42	12	13	23.2	42	11.7	7.7	**M**	11	9	**M**
30	10	18	21.8	30	16	13	17	18.9	17	28	12	20	23.4	28	1.6	4.5	**U**	2	11	**U**
15	10	7	12.6	15	10	12	12	14.0	12	17	8	12	14.6	17	2.0	0.6	**M**	2	5	**U**
38	26	27	37.1	38	22	32	32	35.0	32	25	26	15	26.5	26	10.6	8.5	**M**	12	6	**M**
30	20	20	28.4	30	32	32	32	39.7	32	30	22	20	29.3	30	0.9	10.4	**U**	0	2	**U**
14	9	10	13.4	14	14	9	10	13.4	14	20	6	7	11.7	20	1.7	1.7	**0**	6	6	**0**
45	23	20	34.1	45	35	42	27	42.3	42	35	25	25	34.7	35	0.6	7.6	**U**	10	7	**M**
0	0	0	0.0	0	7	15	7	11.2	15	14	7	3	8.2	14	8.2	2.9	**M**	14	1	**M**
27	24	28	32.6	28	17	25	25	27.3	25	31	24	16	28.3	31	4.3	1.1	**M**	3	6	**U**
17	17	15	20.2	17	14	14	14	17.4	14	22	18	15	22.5	22	2.2	5.1	**U**	5	8	**U**
43	22	18	31.9	43	33	28	34	39.1	34	30	21	25	31.1	30	0.8	8.0	**U**	13	4	**M**
43	20	30	36.7	43	33	33	37	42.5	37	44	20	16	30.0	44	6.7	12.6	**U**	1	7	**U**
0	0	0	0.0	0	0	0	0	0.0	0	0	0	0	0.0	0	0.0	0.0	**0**	0	0	**0**
71	37	35	56.0	71	53	34	52	56.4	53	70	35	36	55.2	70	0.8	1.1	**U**	1	17	**U**
42	29	39	44.9	42	41	40	26	43.3	41	42	35	14	34.0	42	10.9	9.3	**M**	0	1	**U**
46	26	46	47.2	46	50	28	25	40.6	50	55	35	43	54.1	55	6.9	13.5	**U**	9	5	**M**
18	11	14	17.4	18	17	17	16	20.7	17	32	18	16	26.0	32	8.6	5.3	**M**	14	15	**U**

17	11	10	15.3	17	12	16	7	13.7	16	15	13	6	13.1	15	2.2	0.6	**M**	2	1	**M**
0	0	0	0.0	0	0	0	0	0.0	0	0	0	0	0.0	0	0.0	0.0	**0**	0	0	**0**
40	35	27	41.6	40	14	14	14	17.4	14	38	15	20	27.9	38	13.7	10.6	**M**	2	24	**U**
22	20	20	25.6	22	35	22	12	26.0	35	33	25	20	31.6	33	6.0	5.6	**M**	11	2	**M**
41	23	24	35.1	41	38	35	47	49.2	47	45	35	20	39.2	45	4.1	10.1	**U**	4	2	**M**
48	34	35	47.8	48	38	40	45	50.7	45	45	40	40	51.6	45	3.8	0.9	**M**	3	0	**M**
31	24	26	33.3	31	35	25	25	34.7	35	40	20	25	33.7	40	0.4	1.0	**U**	9	5	**M**
40	42	29	45.3	42	42	30	35	43.8	42	40	30	30	41.0	40	4.3	2.9	**M**	2	2	**0**
55	58	36	60.3	58	45	50	33	52.1	50	65	75	70	86.7	75	26.4	34.6	**U**	17	25	**U**
49	27	36	45.0	49	50	36	37	50.3	50	45	40	40	51.6	45	6.6	1.3	**M**	4	5	**U**
23	17	16	22.9	23	18	18	30	26.5	30	36	24	20	32.1	36	9.2	5.6	**M**	13	6	**M**
22	18	16	23.0	22	56	27	20	38.7	56	36	30	17	32.7	36	9.8	5.9	**M**	14	20	**U**
46	42	19	41.2	46	40	38	20	38.7	40	43	35	15	35.1	43	6.2	3.7	**M**	3	3	**0**
22	14	25	24.5	25	62	36	30	50.4	62	0	0	0	0.0	0	24.5	50.4	**U**	25	62	**U**
33	16	26	29.7	33	21	17	17	22.6	21	32	17	20	27.5	32	2.2	4.9	**U**	1	11	**U**

[1] substitutive diameter calculated using formula (1); [2] maximal size of the tumor; [3] **U** - smaller measurement difference between ultrasonography and histology than between MRI and histology, **M** - smaller measurement difference between MRI and histology than between ultrasnography and histology, **0** - the same measurement difference between ultrasound or MRI and histology.

Table S3. Accuracy of ultrasonography in predicting cervical cancer FIGO 2018 stage. (N = 58).

		Histology							
		IA2	IB1	IB2	IB3	IIA1	IIA2	IIB	IVA
**Ultrasonography**	IA2	**0**	0	0	0	0	0	0	0
	IB1	0	**13**	5	0	0	0	0	0
	IB2	0	1	**15**	1	0	0	0	0
	IB3	0	0	4	**12**	0	0	0	0
	IIA1	0	0	0	1	**0**	0	0	0
	IIA2	0	0	0	0	0	**0**	0	0
	IIB	0	0	0	0	0	0	**0**	0
	IVA	0	0	0	0	0	0	0	**0**

Accuracy: 68.97%.

Table S4. Accuracy of MRI in predicting cervical cancer FIGO 2018 stage. (N = 50).

		Histology							
		IA2	IB1	IB2	IB3	IIA1	IIA2	IIB	IVA
**MRI**	IA2	**0**	0	0	0	0	0	0	0
	IB1	1	**14**	5	0	0	0	1	0
	IB2	0	0	**4**	1	0	0	0	0
	IB3	0	0	2	**2**	0	0	0	0
	IIA1	0	0	2	1	**0**	0	0	0
	IIA2	0	0	1	4	0	**0**	0	0
	IIB	0	0	5	2	0	1	**1**	0
	IVA	0	0	2	0	0	0	0	**0**

Accuracy: 42.00%.

Table S5. Accuracy of ultrasonography in predicting cervical cancer T stage in TNM system. (N = 58).

		Histology						
		1a2	1b1	1b2	2a1	2a2	2b	>2b
**Ultrasonography**	1a2	**0**	0	0	0	0	0	0
	1b1	1	**34**	1	0	0	2	0
	1b2	0	4	**12**	0	1	2	0
	2a1	0	0	0	**0**	0	0	0
	2a2	0	0	1	0	**0**	0	0
	2b	0	0	0	0	0	**0**	0
	>2b	0	0	0	0	0	0	**0**

Accuracy: 79.31%.

Table S6. Accuracy of MRI in predicting cervical cancer T stage in TNM system. (N = 50).

		Histology						
MRI		1a2	1b1	1b2	2a1	2a2	2b	>2b
	1a2	**0**	0	0	0	0	0	0
	1b1	1	**23**	1	0	0	1	0
	1b2	0	2	**2**	0	0	0	0
	2a1	0	2	1	**0**	0	0	0
	2a2	0	1	4	0	**0**	1	0
	2b	0	5	2	0	1	**1**	0
	>2b	0	2	0	0	0	0	**0**

Accuracy: 52.00%.

Table S7. Accuracy of ultrasonography in predicting cervical cancer ontogenetic T stage (oT) (N = 58).

		Histology		
**Ultrasonography**		oT1	oT2	oT3
	oT1	**46**	7	0
	oT2	0	**5**	0
	oT3	0	0	**0**

Accuracy: 87.93%.

Table S8. Accuracy of MRI in predicting cervical cancer ontogenetic T stage (oT) (N = 50).

		Histology		
**MRI**		oT1	oT2	oT3
	oT1	**28**	2	0
	oT2	11	**7**	0
	oT3	2	0	**0**

Accuracy: 70.00%.
